# Renshen (Panax ginseng) and Huanglian (Rhizoma Coptidis) For T2DM: A protocol of systematic review and meta-analysis of randomized clinical trials

**DOI:** 10.1097/MD.0000000000023743

**Published:** 2021-01-15

**Authors:** Shengnan Wang, Rensong Yue, Xiaoying Huang, Linzhi Li, Chenyi Xu, Longyan Liu

**Affiliations:** a Hospital of Chengdu University of Traditional Chinese Medicine, Chengdu, ichuan; b Chengdu University of Traditional Chinese Medicine, Chengdu; c GuangXi University of Chinese Medicine, Nanlin, Guangxi, China.

**Keywords:** Huanglian (Rhizoma Coptidis), meta-analysis and systematic review, protocol, Renshen (Panax ginseng), T2DM

## Abstract

**Introduction:**

Type 2 diabetes mellitus (T2DM) is a metabolic disease characterized by high blood sugar caused by impaired insulin action. With an increasing incidence year by year, it has become a worldwide epidemic. Because of its serious, long-term condition, T2DM has a bad impact on the life and well-being of individuals, families and society. Renshen and Huanglian or compound prescription contain Renshen and Huanglian for treatment of T2DM has already been confirmed. However, due to the lack of evidence, there is no specific method or suggestion, so it is necessary to carry out systematic evaluation on Renshen and Huanglian and provide effective evidence for further research.

**Methods and analysis:**

We will search English databases (PubMed, Embase, Web of Science, Nature, Science on line, the Cochrane Library) and Chinese databases (CNKI, Wan Fang, VIP, Chinese biomedical database), from the establishment of database to October 2020, for randomized controlled trials (RCTs) of ginseng and coptis and the compound containing ginseng and coptis in the treatment of T2DM. Primary outcomes: fasting blood-glucose (FBG), 2 Hours Postprandial Blood Glucose (2hPBG), Glycosylated hemoglobin A1c (HbA1c). Additional outcomes: Low Density Lipoprotein (LDL), High Density Lipoprotein (HDL), triglycerides (TG), total serum cholesterol (TC). Two researchers independently extracted the data and evaluated the quality of the included research, and meta-analysis was conducted on the included data using the software of RevMan5.3 and Stata V.12.0.

**Results:**

The results of this study will systematically evaluate the effectiveness and safety of Renshen and Huanglian intervention for people with T2DM.

**Conclusion:**

The systematic review of this study will summarize the current published evidence of Renshen and Huanglian or compound prescription contain Renshen and Huanglian for the treatment of T2DM, which can further guide the promotion and application of it.

**Ethics and dissemination:**

This study is a systematic review; the outcomes are based on the published evidence, so examination and agreement by the ethics committee are not required in this study. We intend to publish the study results in a journal or conference presentations.

**Open Science Framework (OSF) registration number:**

October 18, 2020. osf.io/8gz7c (https://osf.io/8gz7c).

## 1 Introduction

Type 2 diabetes, also called non-insulin-dependent diabetes, takes the abnormality of pancreatic β-cell functions as the core link whose clinical manifestations are Polydipsia, polyphagia, polyuria, weight loss, and fatigue.^[1]^ With the development of society and economy, changes in peoples lifestyles (increased energy intake and decreased exercise, etc.) and the aging of the population, the incidence of type 2 diabetes is increasing year by year globally. The worldwide prevalence of type 2 diabetes mellitus (T2DM) in adults has increased from −150 million affected people in 2000 to >450 million in 2019 and is projected to rise further to −700 million by 2045.^[2]^ Nowadays diabetes has now become the third non-communicable disease that threatens peoples health and lives after cardiovascular disease and tumors.^[4]^

In terms of treatment, the conventional treatments for T2DM are insulin therapy and oral hypoglycemic medications.^[3]^ Meanwhile regular exercise and a well-controlled diet are essential for the management of type 2 diabetes.^[5]^ However, existing medication therapies can cause gastrointestinal side effects such as hypoglycemia, flatulence and diarrhea, as well as adverse reactions such as cardiovascular diseases, which will seriously affect the quality of life of patients.^[6]^

In recent years, traditional Chinese medicine has been widely used in clinical and experimental study of T2DM, which had been fully proven effective. Renshen and Huanglian are botanical herbs commonly used in traditional Chinese medicine and the exploration of diabetes treatment methods show that Renshen and Huanglian had good effects on the controlling of blood sugar and improvement of diabetes symptoms,^[7]^ but its effectiveness and safety have not yet reached a definitive conclusion. Therefore, this research intends to adopt the method of system valuation and meta-analysis of Renshen and Huanglian or contain Renshen and Huanglian formula in the treatment of type 2 diabetes to evaluate the efficacy and safety.

## 2 Methods

### 2.1 Study registration

The protocol has been registered in OSF (Open Science Framework) Preregistration. October 18, 2020. osf.io/8gz7c (https://osf.io/8gz7c). The protocol will follow the statement guidelines of Preferred Reporting Items for Systematic Reviews and Meta-Analyses Protocols (PRISMAP),^[8]^ Changes will be reported in the full review as required.

### 2.2 Inclusion and exclusion criteria for study selection

#### 2.2.1 Inclusion criteria

Inclusion criteria are all randomized controlled trials (RCTs), Which treatment of T2DM are Renshen and Huanglian serves as 2 herbs or the 2 herbs are main elements in mixture herb formulas. The language of the trials to be included only Chinese or English.

#### 2.2.2 Exclusion criteria

Following studies will be excluded:

1. patients age <18 years old
2. other types of diabetes. Such as Type 1 diabetes, Gestational diabetes and so on.
3. The treatment was combined with other treatment other than Chinese herbs.
4. Non-RCTs and Quasi-RCTs
5. Case series and Reviews
6. Animal studies

### 2.3 Types of participants

Types of participants included people diagnosed with T2DM, no matter the degree and possible complications. All the patients should be treated by traditional Chinese medicine included 2 herbs Renshen and Huanglian, or the 2 herbs are main elements in mixture herb formulas, or the 2 herbs combine with other conventional treatments. No sex, ethnicity, or education restriction is here.

### 2.4 Experimental interventions

The traditional Chinese medicine Renshen and Huanglian should be the main treatments.

### 2.5 Control interventions.

Interventions may include: No treatment, the placebo, Non-drug interventions (e.g., diet, exercise, etc.), Conventional western medicine hypoglycemic drugs (e.g., metformin, euglycemic, etc.), Insulin (any kind of insulin). Combined interventions are allowed if all groups in the randomized trial receive the same combined intervention.

### 2.6 Types of outcome measures

#### 2.6.1 Main outcomes

1. Glycosylated hemoglobin A1c (HbA1c);
2. fasting blood-glucose (FBG);
3. 2 Hours Postprandial Blood Glucose (2hPBG).

#### 2.6.2 Additional outcomes

1. Low Density Lipoprotein (LDL)
2. High Density Lipoprotein (HDL)
3. triglycerides (TG)
4. total serum cholesterol (TC).

## 3 Data sources

### 3.1 Electronic searches

The following data bases will be searched to identify eligible studies: PubMed, Embase, Cochrane Library, Web of Science, Nature, Science on line, Chinese Biomedical Database WanFang, VIP medicine information, and CNKI (China National Knowledge Infrastructure). The time range is: the starting time is determined according to the first literature available, and the deadline is October 2020.

### 3.2 Other search resources

**Figure 1:**
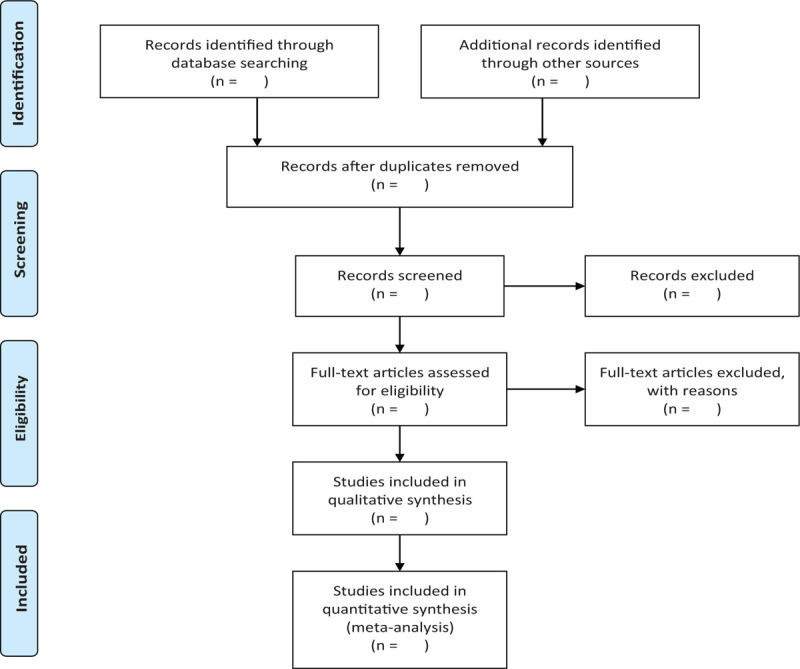
The reasearch flowchart.

In order to get more complete evidence, we will also retrieve other related documents by manually, such as medical textbooks, clinical laboratory manuals and so on. If it is necessary to contact with trail author to obtain the latest clinical data, we will do it. Moreover, studies associated with the review will be identified via evaluating related conference proceedings. The research flowchart is shown in Figure 1.

### 3.3 Search strategy

The following search terms will be used: randomized controlled trial/RCT; type 2 diabetes/T2DM; traditional Chinese medicine/TCM; Renshen/ Huang-lian/Huanglian/coptis, different retrieval strategies in Chinese and foreign databases will be used. Language restrictions are Chinese and English. There is no publication restriction. Here we take the search strategy in PubMed as an example and list in Table 1.

**Table 1:** Search stragtegy sample of PubMed.

number	search
#1	Diabetes Mellitus, Type 2 (MeSh)
#2	Diabetes Mellitus, Type 2 (ti,ab)
#3	T2DM (ti,ab)
#4	Type two diabetes Mellitus (ti,ab)
#5	or#1-4
#6	Medicine, Chinese Traditional (MeSh)
#7	Traditional Chinese medicine (ti,ab)
#8	TCM (ti,ab)
#9	Or 6–8
#10	Panax (MeSh)
#11	Panax (ti,ab)
#12	Ginseng (ti,ab)
#13	Renshen (ti,ab)
#14	Ren-shen (ti,ab)
#15	Or#10–14
#16	Coptidis (MeSh)
#17	Rhizoma Coptidis (ti,ab)
#18	Coptidis (ti,ab)
#19	Huanglian (ti,ab)
#20	Huang-lian (ti,ab)
#21	Or#15-20
#22	Randomized Controlled Trial (MeSh)
#23	Randomized Controlled Trial (ti,ab)
#24	RCT (ti,ab)
#25	#16–19
#26	#5 and #9 and #15 and #21 and #26

## 4 Data collection and analysis

### 4.1 Study selection

All articles in the search results were independently evaluated by 2 independent researchers (WS, YR) according to inclusion and exclusion criteria. Reviewers will then independently extract and collect the data included in the study using pre-designed data collection forms. Discrepancies will be discussed and resolved by consensus with a third author (HX).

### 4.2 Data extraction and management

The following information will be extracted from each study:

1. Normal test characteristics: title, author, year.
2. baseline data: sample size, age, gender, diagnostic criteria, course of disease.
3. interventions: dosage of Huanglian and Huangqin, control of intervention details, intervention.

If the information is not enough, we will contact experts and authors in this field to get relevant information.

### 4.3 Assessment of the reporting quality and risk of bias

The risk of bias will be assessed by 2 independent authors (HX and WS), together with completing the STRICTA checklist.^[9]^ The Cochrane System Evaluator's Manual give the evaluation criteria for authors to evaluated the RCTs quality. Assessing the risk of bias:

1. random sequence generation;
2. allocation concealment;
3. blinding of participants and personnel;
4. blinding of outcome assessment;
5. incomplete outcome data;
6. selective outcome reporting;
7. other bias.

Any disagreement will be discussed or consulted with a third reviewer. Each them will be described from 3 levels: “high risk”, “low risk”, and “unclear”.

### 4.4 Measures of a treatment effect

The dichotomous outcomes will be expressed by the Odds ratio (ORs), while the continuous data will use the standardized mean difference (SMD). All these outcomes report 95% CIs.

### 4.5 Management of missing data

We will take the method of contacting corresponding authors to obtain the missing data. If there is no response, we will analyze only the available data and describe the reason and impact of this exclusion in the paper.

### 4.6 Assessment of a reporting bias

The bias of publication will be explored through funnel plot analysis. If the funnel plot show asymmetry, it will be evaluated via the Egger and Begg tests, and *P* value <.05 means the publication bias is significant.

### 4.7 Assessment of heterogeneity

There are 2 main methods for testing heterogeneity, namely graphical method (funnel plot, forest plot) and statistical test (Q value statistic test, *I*^2^ statistic test, H statistic test). The *I*^2^ statistic test method shows us When *I*^2^ is 0, it means that studies are completely homogeneous, If *I*^2^ > 50%, it indicates there is heterogeneity in studies.

### 4.8 Data synthesis and grading of quality of evidence

The results of the study will be analyzed by RevMan 5.0 software provided by Cochrane collaborate on network. The binary data will be expressed by the odds ratio, while the continuous data will use the mean difference (MD). To test the heterogeneity of the research results, when the *I*^2^ < 50% or *P* > .1, the heterogeneity is significant. The random effect model was used for the meta-analysis, otherwise, we choose the fixed effect model.

### 4.9 Subgroup analysis

When the heterogeneity test results are heterogeneous, we need to clarify the source of the heterogeneity by subgroup analysis. The effects of different types of therapy including design scheme, severity of illness, age, sex, and mild or severe T2DM were analyzed. We will also delete low-quality and/or medium-quality studies to check the robustness of the results.

### 4.10 Sensitivity analysis

Sensitivity analysis can not only assess the stability and reliability of the conclusions of the meta-analysis, but also assess whether the changes in the results are related to the impact of a single study. If the stability of the conclusion is poor, we can achieve the purpose of increasing stability by changing the analysis model, inclusion criteria and exclusion criteria, or excluding a certain type of literature.

### 4.11 Ethics and dissemination

We will publish the system review results in peer-reviewed journals, disseminated in meetings or in peer-reviewed publications. Aggregated published data will be used to excluded data of individuals, so there is no need for obtaining the ethical approval or patients informed consent.

## 5 Discussion

In Traditional Chinese medicine, ginseng, with the function of supplementing qi and producing saliva,^[10]^ is often used for a variety of debilitating diseases. Modern pharmacological studies have shown that ginseng can regulate immunity, improve micro-circulation and regulate blood sugar, ect.^[11]^ Ginseng mainly contains active ingredients such as Rg1, Re, Rb1, Rc, Rg2, Rb2, Rb3, and Rd and so on. Among them, ginsenoside is the main effective ingredient in the treatment of diabetes, which can be used for diabetes and various chronic complications. Its mechanism of action may be related to the promotion of GLP-1 secretion. Secondly, the active ingredient Rg1 can play a hypoglycemic role by inhibiting glucose absorption in the small intestine and glucose hetero-genesis in the liver.^[12]^

Coptis chinensis has the effect of clearing away heat and detoxifying, and its modern pharmacological effects mainly include anti-oxidation, anti-virus, regulation of positive inotropic action, anti-hypertension, glucose-lowering, lipid-lowering and anti-inflammatory.^[11]^ Berberine is the main effective component of coptis chinensis in the treatment of diabetes mellitus. Its therapeutic effects include: promoting the repair and regeneration of pancreatic beta cells; activating insulin receptor gene expression, increasing peripheral tissue sensitivity to insulin^[13]^; reliefing insulin resistance, promoting the secretion of glucagon - like peptide 1 (GLP-1) and insulin, attenuating intestinal inflammation, protecting intestinal mucosal barrier, promoting glucose transport, and reducing the level of peripheral blood glucose.

T2DM belongs to the category of “Xiaoke” in Traditional Chinese medicine. The pair herbs of ginseng and coptis Chinensis or the formulas contain ginseng and coptis Chinensis are mostly used to treat “Xiaoke” in Traditional Chinese medicine, which has achieved good clinical efficacy.^[14]^ Studies have shown that ginseng and coptis have definite effect on the treatment of T2DM, and the effect is related to promoting drug metabolism, improving insulin resistance, and improving inflammatory response, etc. The possible biological mechanisms mainly include: AMPK signaling pathway, NF-κB signaling pathway, TNF signaling pathway and glucagon signaling pathway.^[13]^ Ginsenoside Re and berberine are the main effective components to improve diabetes, respectively. Studies have confirmed that both of them can cure insulin resistance and disorder of glucose and lipid metabolism in diabetic mice.^[9,14,15]^ When the ratio of ginsenoside Re to berberine was 4:16 (100 μg/g), it showed more significant hypoglycemic effect^[9,16]^ and inhibition of α-glucosidase^[16]^ Studies have shown that the hypoglycemic mechanism of rhizoma coptidis and ginseng herb pairs may have the following aspects:

1. Up-regulate the expression of ADPNR1, improve the bioavailability of ADPN, or regulate the Notch1-NGN3^[16]^ signaling pathway, improve islet fibrosis, and promote the repair of islet damage^[17]^;
2. Regulate the levels of FBG, NIS, FFA and TNaF in T2DM model animals, improve insulin resistance,^[15,18]^ and enhance insulin sensitivity, which is similar to the common hypoglycemic oral drug pioglitazone;
3. Reduce the level of inflammatory factors in diabetic rats, down-regulate the expression of inflammatory factors and pro-apoptotic factors, inhibit cell apoptosis, and promote the repair of islet cell injury.^[19]^

In conclusion, the systematic review and meta-analysis are helpful to determine the potential value of Renshen and Huanglian or the 2 herbs combination therapy for T2DM. This study can not only provide the basis for the release of diabetes treatment guidelines, but also promote the application of traditional Chinese medicine prescriptions, so that more patients benefit.

## Author contributions

**Conceptualization:** Shengnan Wang, Xiaoying Huang, Rensong Yue.

**Data curation:** Linzhi Li; Chenyi Xu.

**Formal analysis:** Shengnan Wang, Xiaoying Huang.

**Funding acquisition:** Rensong Yue.

**Investigation:** Chenyi Xu.

**Methodology:** Shengnan Wang, Xiaoying Huang, Rensong Yue.

**Project administration:** Rensong Yue.

**Resources:** Shengnan Wang, Longyan Liu, Linzhi Li.

**Software:** Shengnan Wang, Xiaoying Huang.

**Supervision:** Rensong Yue.

**Visualization:** linzhi Li.

**Writing – original draft:** Shengnan Wang

**Writing – review & editing**: Rensong Yue.

## Abbreviations

Abbreviations: CI = confidence interval, CNKI = China National Knowledge Infrastructure, EBM = evidence-based medicine, Huanglian = Rhizoma Coptidis, Huangqin = Scutellaria, RCT = randomized controlled trial, RR = relative risk, SR = systermatic review, WMD = weight mean difference.
